# Plasmonic Spectral Splitting in Ring/Rod Metasurface

**DOI:** 10.3390/nano7110397

**Published:** 2017-11-19

**Authors:** Naseer Muhammad, Adnan Daud Khan, Zi-Lan Deng, Karim Khan, Ashish Yadav, Qiang Liu, Zhengbiao Ouyang

**Affiliations:** 1 THz Technical Research Center of Shenzhen University, Shenzhen Key Laboratory of Micro-Nano Photonic Information Technology, Shenzhen 518060, China; naseer@szu.edu.cn (N.M.); karim_khan_niazi@szu.edu.cn (K.K.); ashish84yadav@gmail.com (A.Y.); qliu@szu.edu.cn (Q.L.); 2 Key Laboratory of Optoelectronics Devices and Systems of Ministry of Education and Guangdong Province, Shenzhen 518060, China; 3 College of Electronic Science and Technology, Shenzhen University, Shenzhen 518060, China; 4 Department of Electrical Engineering, Sarhad University of Science and Information Technology, Peshawar 25000, Pakistan; adnandaudkhan@gmail.com; 5 Guangdong Provincial Key Laboratory of Optical Fiber Sensing and Communications, Guangzhou 510632, China; zilandeng@jnu.edu.cn; 6 Institute of Photonics Technology, Jinan University, Guangzhou 510632, China

**Keywords:** *Q*-factor, figure of merit, fano resonance, finite element method, sensors, nanostructures

## Abstract

We report spectral splitting behaviors based on Fano resonances in a novel simple planar metasurface composed of gold nanobars and nanorings. Multiple plasmonic modes and sharp Fano effects are achieved in a broadband transmittance spectrum by exploiting the rotational symmetry of the metasurface. The transmission properties are effectively modified and tuned by modulating the structural parameters. The highest single side *Q*-factor and FoM which reaches 196 and 105 are observed at Fano resonances. Our proposed design is relatively simple and can be applied for various applications such as multi-wavelength highly sensitive plasmonic sensors, switching, and slow light devices.

## 1. Introduction

The collective oscillations of electrons in metallic nanostructures driven by an external electromagnetic field known as “surface plasmons” have received much attention at present due to their potential applications in biomedical imaging [1], sensing [2], cancer therapeutics [3], surface-enhanced spectroscopies [4,5], Salisbury screens [6,7], and energy harvesting [8]. To achieve plasmonic modes with higher quality factors (*Q*-factors) [9] and lower radiative losses [10], one has to excite multiple modes in a plasmonic nanoparticle [11,12,13,14]. Two approaches have been reported for the excitation of multiple modes: (1) using a cluster of nanoparticles, where the individual resonant plasmon modes of the nanoparticles couple to each other and split the optical spectrum into multiple modes [15,16,17,18], (2) symmetry breaking, which has been investigated in single [19] as well as in cluster of nanoparticles [20]. The newly excited modes (which were dark before) can interact with the bright modes and induce plasmonic Fano-like resonances, which can be used for lasing, switching and slow light applications [21,22], and terahertz sensing [23,24]. These multiple modes and Fano resonances extremely depend upon the geometry of the nanoparticle, size, and refractive index of the embedding medium [25]. A variety of nanostructures supporting multiple modes have been investigated. Hu et al., presented gold-silica-gold multilayered nanoshell, where the structure’s symmetry is relaxed by offsetting the inner gold core due to which extinction spectra split to have multiple modes [26]. Dayal et al., reported high *Q*-factor (*Q* = 70–80) Fano resonances in plasmonic metasurfaces in the infrared region [9,27,28]. Khan et al., proposed a dimer based on bimetallic multilayered nanoshell, where multiple modes are excited by displacing the inner core and middle silica layer of both the nanoparticles simultaneously [29]. Zhang et al., investigated heptamer clusters composed of asymmetric split ring resonators [30]. The dipole and quadrupole modes of each split ring couple and induce multiple Fano resonances in the extinction spectra. Liu et al. illustrated a polarization-independent plasmonicnonamer cluster comprised of a nanocross surrounded with eight nanorods that exhibit higher order modes in the visible region and multiple Fano resonances in the near infrared region [31]. However, the nanostructures reported to date are complex and require complex fabrication techniques. Moreover, the bulky metamaterials suffer from large ohmic losses due to which they exhibit Fano resonances with small modulation depths in the optical spectrum [32,33]. Thanks to advanced fabrication techniques, one can fabricate a small structure of a few nanometers precisely, which can yield sharp spectral features [9].

In this paper, Fano resonances with spectral splitting are numerically investigated in simple and novel compact planar metasurface composed of a gold nanobar and a gold nanoring. Coherent coupling between the two structures results in multiple sharp Fano resonances in the broadband transmission spectrum. Compared to the previous relevant art [34,35,36,37], in our designed structure highly interesting and sharp spectral signatures are investigated. Such multiple resonances are effectively tuned by changing the parameters of the metasurface. The proposed metasurface may pave a new manner for the design of multi Fano-like spectrum splitter.

## 2. Physical Module

**Figure 1:**
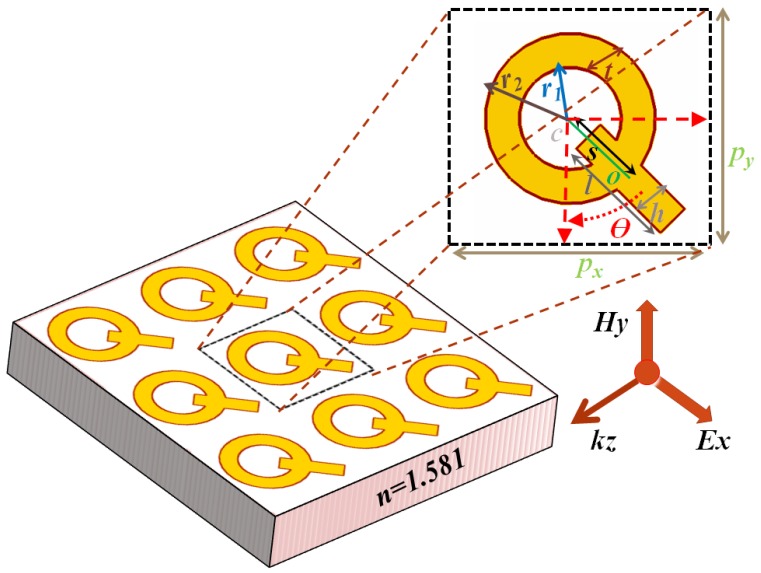
Schematic of the ring/rod gold metasurface.

The array of ring/rod metasurface placed on a dielectric substrate is shown in Figure 1. Floquet boundary conditions as periodic boundary conditions are used to repeat the unit cell with a period of *p* = 180 nm along *x*- and *y*-direction as shown in the inset in Figure 1. The structure parameters are: the radius of the outer ring *r*_2_ = 50 nm, the radius of the inner ring *r*_1_ = 30 nm, the thickness of the ring *t* = 20 nm, the length of nanorod *l* = 70 nm, the width of the nanorod *h* = 20 nm, the thickness of gold material in −*z* to *z* direction is 20 nm, the angle of rotation of nanorod in the clockwise direction θ, the center of the ring *c*, the center of nanorod *o*, and the distance *s* between the center of the ring *c* and that of the nanorod, respectively. The structure is designed on the surface of 490 nm silica substrate having refractive index of *n* = 1.581. The nanostructure is embedded in air and is excited by *x*-polarized (0° incident angle) electromagnetic beam propagating in the *z*-direction, with unity power. The transmission properties of the metasurface are carried out by COMSOL Multiphysics 5.1 software with RF module. A Johnson and Christy data model has been used for the dielectric constant of the gold [38].

## 3. Results and Discussion

**Figure 2:**
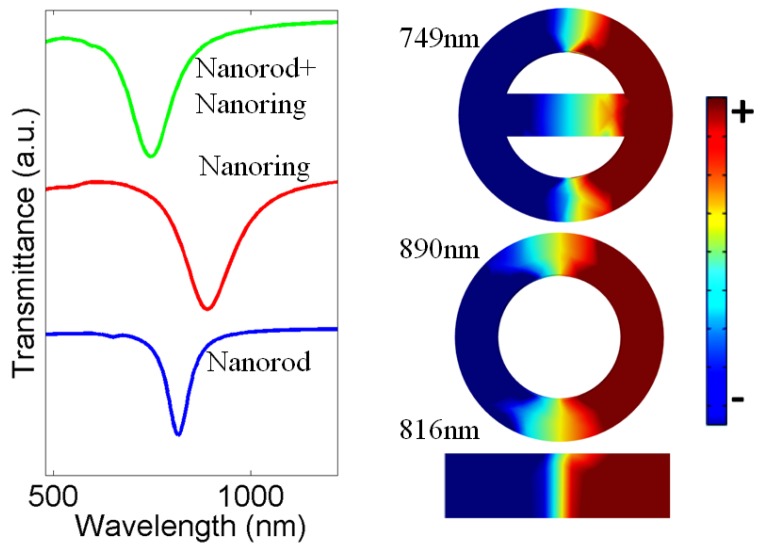
Transmittance spectra of nanorod (blue curve), nanoring (red curve), compact ring/rod (green curve) structures. Inset shows surface charge distributions of all the structures at their resonant wavelengths. Where red indicates positive and blue indicates negative.

We first considered the transmittance characteristics of the nanorod and nanoring, which is shown by the blue and red curves in Figure 2. The nanorod is placed along the direction of polarization, so efficient coupling will occur between the incident light and nanorod. Thus, the mode obtained at 816 nm is a bright dipole mode, which is also disclosed by the surface charge distributions shown in the inset in Figure 2. The red curve reveals the transmittance spectra of the nanoring, where again a broad dipole mode at 890 nm is obtained. The resonance wavelength of the nanoring is longer than that for the nanorod as the nanoring has a larger effective dielectric constant (as the effective width of the nanoring is larger than that of the nanorod) and thus a longer resonance effective length than the nanorod. Next, we insert the nanorod, which is placed at 0° or in other words at 360° inside the nanoring and calculated its transmittance spectrum as shown by the green curve. In this case, the metasurface adopts the theta-shaped structure [37]. The mode obtained at 749 nm is a hybridized dipole mode because it arises from the coupling of dipole modes of the nanorod and nanoring structures. It can also be regarded as arisen from the coupling of modes in the upper and lower half circles in the theta structure. Obviously, the half-circle structure has the smallest effective width and thus smallest effective dielectric constant in the three structures because the half-circle structure consists of a half nanoring and half side of the nanorod, and the nanorod in the theta structure is shorter than that of the free nanorod. Therefore, the resonance wavelength of the half-circle structure is the shortest for the three structures, while the resonance wavelength of the theta structure is approximately that of the half-circle structure. The dipole nature of the mode is also revealed by the surface charge distributions as shown in the inset, where both the nanoring and nanorod exhibit a dipolar pattern.

**Figure 3:**
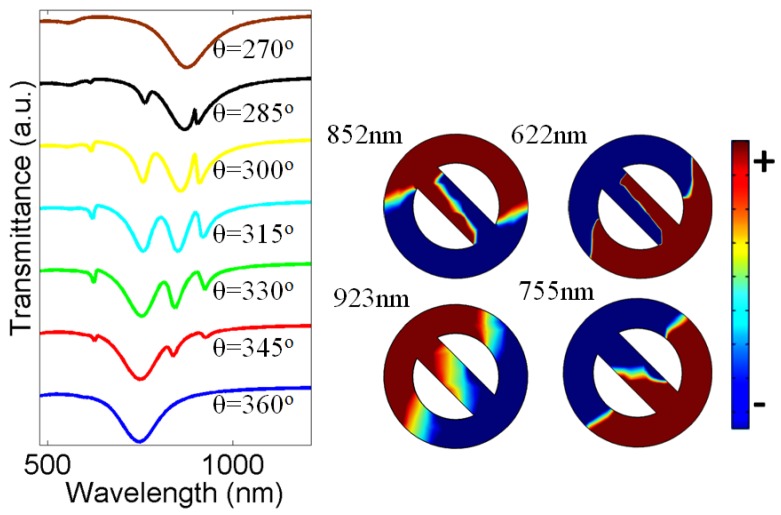
Transmittance spectra of theta-shaped metasurface at different values of θ. Inset shows surface charge distributions at θ = 315°.

Now to split the transmittance spectrum of the theta-shaped metasurface, we rotate the nanorod in a clockwise direction. In this way, the effective dielectric constant of the nanorod will change, which further cause variation only in the effective refractive index of the nanorod. However, the effective refractive index of the nanoring does not change. Therefore, the wavelength of the resonant mode resulting from the nanorod will change, while the resonant wavelength yields from the nanoring will not change. As a result, Fano resonance due to the interference of the two modes with a small difference in frequency will occur. Figure 3 shows the transmittance characteristics of the theta-shaped structure for different values of θ. When we vary the angle from 360° to 345°, modes of different wavelengths supported by the nanorod and nanoring start coupling with each other yields the transmittance spectrum split and generate multiple modes. The intensity of modes increases by further rotating the nanorod in a clockwise direction. For instance, at θ = 315°, the newly excited modes look very prominent. To understand the nature of the modes, we calculated the surface charge density distributions as shown in the inset in Figure 3. All the modes show rotated dipoles because, when we rotate the nanorod, the dipole also rotates. It can be noted that the line shapes of the two newly excited modes at the lower energy side appear highly asymmetric, which represents Fano-like resonances. These Fano resonances emerge due to coupling of the broad mode with the two narrow modes. There is also a small mode located at 622 nm, which also represents a dipole mode. However, this mode is far ahead from the broad dipole mode, therefore coupling will not take place. By further decreasing the rotation angle θ, the intensity of the newly excited modes decreases and eventually disappears at θ = 270° because at this value of θ, the electric vector of the light is parallel to the nano metallic rod and the light will be completely absorbed. So, in this way, we can rotate the dipoles as the transmittance characteristics of the metasurface are extremely sensitive to the angle between the axis of the nanorod and the polarization vector of the incident wave. Such metasurface can be used for switching applications—i.e., to switch Fano resonances on and off. It should be pointed out, from the symmetry of the system it can be understood that the spectra for the angle in the ranges from 360 to 270, from 270 to 180, from 180 to 90, and from 90 to 0 are similar.

From Figure 3, it can be seen that there are three extra dips in the spectrum, it means that the difference between the two wavelengths interfering to have the Fano resonances leads to a phase difference of 3 × π because for each π of phase difference, there can be one Fano resonance mode. Table 1 shows the single side *Q*-factor (*Q_ss_*) and figure of merit (FoM) values calculated around Fano resonances for θ = 300°, 315°, and 330° using the following expressions: *Q_ss_* = 0.5 × λ_max_/(λ_max_ − λ_hp_), where λ_max_ shows the wavelength at maximum power, and λ_hp_ shows the wavelength at half power. FoM = *Q* × ΔI, where *Q* is the quality factor and ΔI the resonance intensity. Quality factor and resonance intensity are two important parameters for sensing [24,39]. The *F*_1_, *F*_2_, and *F*_3_ indicate the first, second, and third (from right to left—i.e, from larger to shorter wavelengths) Fano resonances, respectively. The highest *Q*-factor and FoM values are obtained for θ = 300° (Fano mode *F*_1_), which reaches around 71 and 33, respectively.

**Figure 4:**
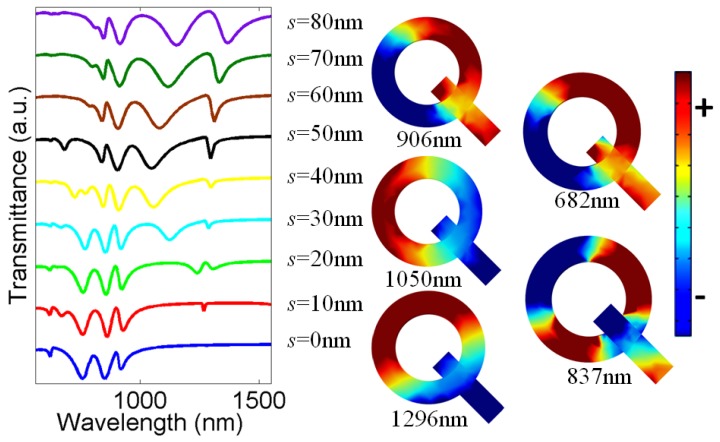
Transmittance spectra of ring/rod metasurface for different values of *s*. Inset shows surface charge distributions at *s* = 50 nm.

Since multiple modes with high intensity are obtained for θ = 315°, therefore, for further analysis, we will only consider this value of θ. Next, we changed the parameter ‘*s*’, which represents the distance between center of the nanoring *c* and center of nanorod *o* and keep all other parameters constant. Changing *s* means that we will gradually move the nanorod outside from the nanoring due to which the theta-shaped structure will be converted into a Q-shaped structure. Figure 4 shows the transmittance properties of the metasurface for different values of *s*. *s* = 0 nm (blue curve), meaning that the center of the nanorod and nanoring are at the same point, so the spectra obtained are already discussed in the previous section. When *s* > 0 nm, the mode shifting occurs and new modes emerge in the spectrum. For instance, at *s* = 50 nm, four different modes can be seen in the spectrum. The surface charge density distributions are calculated to understand the nature of each mode. It appears that all the modes represent rotated dipoles except a small peak around 837 nm represents a quadrupole mode in the ring and dipole mode in the bar. However, this small peak disappears for higher value of *s*. Furthermore, the line shapes of the modes are highly asymmetric, so they also represent Fano resonances. Therefore, like the theta-shaped metasurface, the Q-shaped can also be used for slow light and lasing applications. Thus, the parameter *s* can play an important role in splitting the transmittance spectrum of the metasurface. Table 2 demonstrates the single side *Q*-factor (*Q_ss_*) and FoM values calculated around Fano resonances for *s* = 50, 60, 70, and 80 nm, respectively. The highest *Q*-factor and the corresponding FoM values are calculated for *s* = 50 nm (Fano mode *F*_1_), which are around 64 and 32.

From Figure 4, it can be seen that the resonance wavelength becomes longer as *s* increases. This is because the effective length of the structure becomes longer, i.e., longer cavity and longer resonance wavelength. Furthermore, from Figure 4 it can be seen that there are four to five extra dips in the spectrum except the basic dip. This means that the difference between the two wavelengths interfering to have the Fano resonances leads to a phase difference of (4–5) × π because for each π of phase difference, there can be one Fano resonance mode. Moreover, from Figure 3 and Figure 4, it can be seen that the resonance wavelength for the Q-shaped structure is longer than that for the theta-shaped structure. This can be explained by noting that the Q-shaped structure has a longer effective length than that of theta-shaped structure.

**Figure 5:**
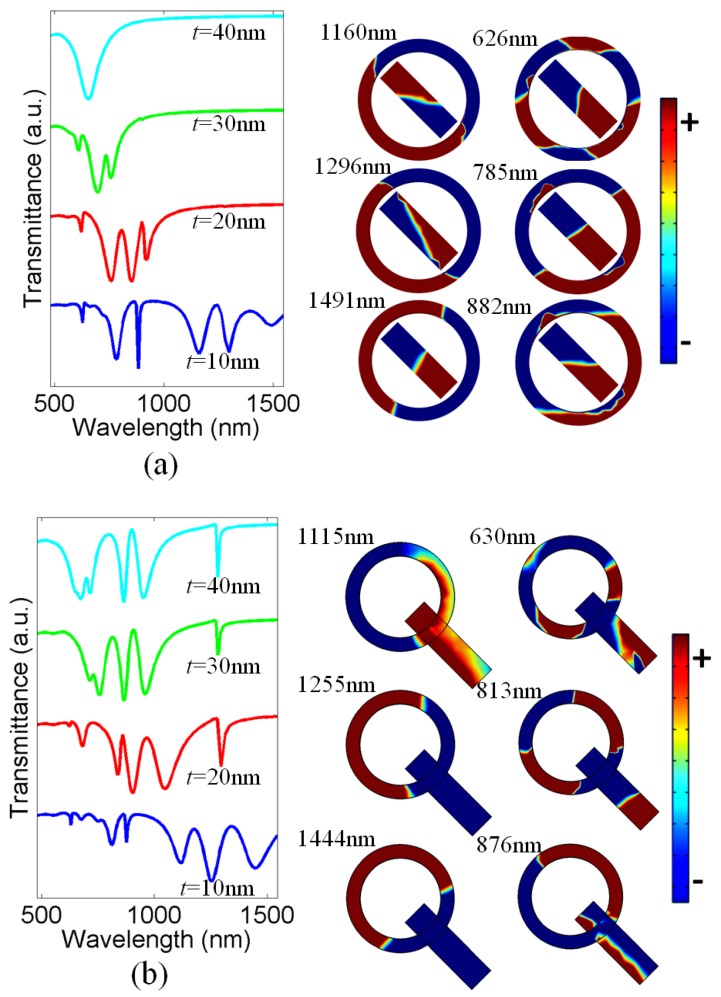
(**a**) Transmission spectra of theta-shaped metasurface. Inset shows surface charge distributions calculated at *t* = 10 nm; (**b**) Transmission spectra of Q-shaped metasurface for different values of *t*. Inset shows surface charge distributions calculated at *t* = 40 nm.

Next, we consider both the theta-shaped and Q-shaped structures and vary the thickness *t* of the nanoring. In both cases, we vary the internal radius and keep the external radius constant at *r*_2_ = 50 nm. Figure 5a shows transmittance properties of theta-shaped metasurface for different values of *t*. It appears that, by reducing the value of *t*, the transmission spectra gradually split into multiple modes. The spectrum characteristic in Figure 5a can be explained as follows: when decreasing *t* of the nanoring by increasing the inner radius of the nanoring, the effective length of nanoring will increase and thus the resonant wavelength of the nanoring will become longer. As a result, smaller *t* implies that larger times of π-phase difference can be generated between the resonance mode in the nanoring and that in the nanorod, and therefore more modes of Fano resonance appear. Furthermore, as *t* decreases, the resonant wavelength of the nanoring becomes longer and thus the Fano resonance modes shift to the longer wavelengths. The surface charge distributions calculated at each resonant mode illustrate that the nature of all the modes are similar, except the mode located at 626 nm shows an octupole pattern on the nanoring due to which this mode is a higher order octupole mode. Moreover, four Fano-like resonances with distinct asymmetric line shapes are also observed. The octupole mode at the low wavelength region will not participate in the formation of Fano resonances.

Figure 5b shows transmittance characteristics of Q-shaped metasurface for different values of *t*. Again, the multiple modes can be tuned and switched on/off by varying *t*. In this case also, six multiple modes—including four different Fano resonances—are realized in the spectrum. The resonant mode shifting for this design is not so large compared to the previous case because the distance, which influences the phase difference, between here the mode supported by the nanorod and that supported by the nanoring is much larger than position shift of the mode on the ring caused by the variation of *t*, so that the response of the Q-shaped structure is not so sensitive to *t* as the theta-shaped structure. The line shapes of the Fano resonances are highly asymmetric and different from each other. Thus, the thickness of the nanoring plays an important role in the generation of multiple modes and Fano effects. Table 3 illustrates the single side *Q*-factor (*Q_ss_*) values and FoM calculated around Fano resonances at *t* = 10, 20, 30, and 40 nm for both theta-shaped and Q-shaped nanostructures. The highest values are obtained throughout the spectrum. The sharp Fano resonances in the context of electromagnetically induced transparency possesses highly dispersive medium [23], which indicates that the proposed design may be used for efficient slow light devices.

**Figure 6:**
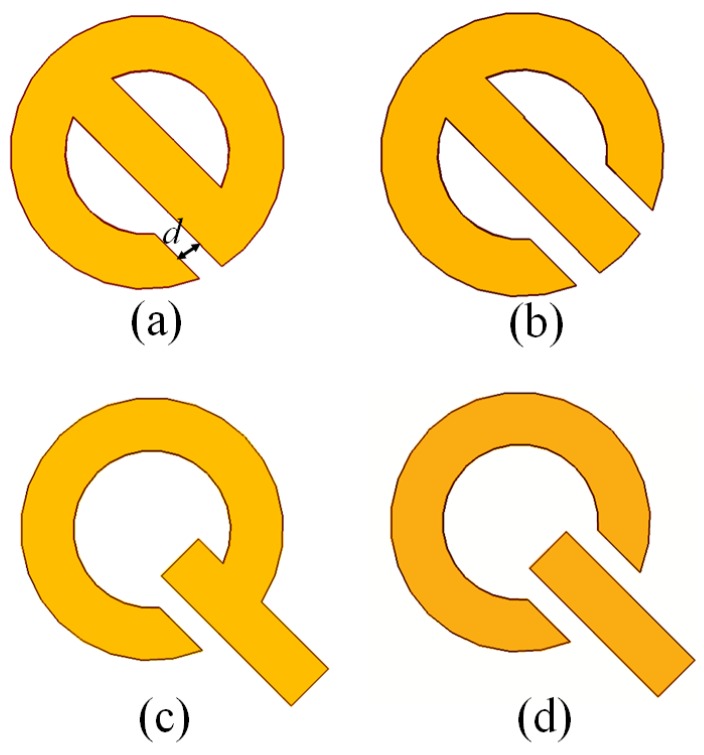

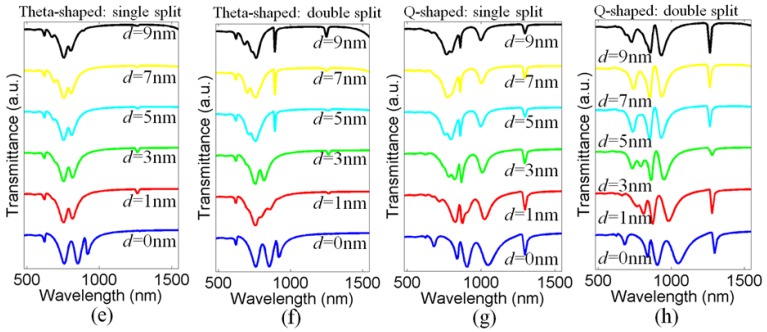
(**a**–**d**) Schematics of theta-shaped and Q-shaped metasurfaces with single and double splits having size “*d*”; (**e**–**h**) Transmittance spectra of both the nanostructures at different values of *d*.

To further modify the transmission properties of the metasurface, we took the best cases from the previous sections and introduced another symmetry-breaking scheme as shown in Figure 6a–d. Here, we incorporate single and dual splits in both the theta-shaped and Q-shaped metasurfaces due to which the plasmon hybridization will occur between the nanoring, nanorod, and cavity modes. Figure 6e,f shows the transmittance characteristics of theta-shaped structure for different values of split size “*d*”. It appears that when *d* > 0 nm, the mode cancellation will take place—i.e., the cavity modes and theta-shaped metasurface modes strongly interfere destructively at many places. Therefore, creating splits in this theta structure is not a better choice. In contrast, the transmittance spectra of the Q-shaped structure given in Figure 6g,h are slightly changed with *d*. These results show that by introducing splits in ring/rod metasurface, no more multiple modes are excited in the transmittance spectra. However, in Q-shaped at larger value of *d* mode cancellation is not so high. This is so because the split is very narrow and thus has small influence to the resonant wavelength of the ring and thus less influence to the interference of the resonant modes. Table 4 reveals the single side *Q*-factor (*Q_ss_*) and FoM values calculated around Fano resonances at *d* = 5, 7, and 9 nm for the theta-shape with a double split, Q-shape with a single split, and Q-shape with a double split. The highest *Q*-factor value in this case is obtained for the Q-shape with a single split which reaches around 117, while the highest value of FoM is calculated for theta-shaped with double splits, which is approximately 71.

## 4. Conclusions

We numerically investigated plasmonic spectral splitter based on Fano resonance effects. By accurately engineering geometrical parameters of the ring/rod metasurface, multiple plasmonic modes and Fano resonances are obtained in the spectrum. The Fano resonance behaviors can be spectrally tuned and modified by properly modulating the structural parameters. The results show that by creating single and double cavity splits in the metasurface, number of plasmonic modes and Fano resonances are essentially reduced. Furthermore, in this work, high *Q*-factor and FoM values are achieved around Fano resonances, which are approximately 196 and 105, respectively. To our knowledge, the multiple modes and sharp Fano features exhibited by our design are not realized in any relatively simple Plasmonic metasurface reported before [37,40].

## Figures and Tables

**Table 1:** *Q*-factor and FoM values around Fano resonances.

Rotation Angle (θ)	300°			315°			330°		
Fano Modes	*F*_1_	*F*_2_	*F*_3_	*F*_1_	*F*_2_	*F*_3_	*F*_1_	*F*_2_	*F*_3_
*Q*-factor	71	29	48	39	17	15	49	26	11
FoM	33	17	16	13	11	7	14	11	6

**Table 2:** *Q*-factor and FoM values around Fano resonances.

S	50 nm			60 nm			70 nm			80 nm		
Fano Modes	*F_1_*	*F_2_*	*F_3_*	*F_1_*	*F_2_*	*F_3_*	*F_1_*	*F_2_*	*F_3_*	*F_1_*	*F_2_*	*F_3_*
*Q*-factor	64	12	18	56	11	20	35	14	12	17	8	18
FoM	32	6	10	35	7	12	24	9	7	13	6	11

**Table 3:** *Q*-factor and FoM values around Fano resonances.

Theta-shaped	*T*	10 nm				20 nm				30 nm				40 nm		
	Fano Modes	*F*_1_	*F*_2_	*F*_3_	*F*_4_	*F*_1_	*F*_2_	*F*_3_	*F*_4_	*F*_3_	*F*_2_	*F*_3_	*F*_4_	*F*_1_	*F*_2_	*F*_3_
	*Q*-factor	25	16	113	17	44	20	19	x	45	19	35	x	8	x	x
	FoM	12	8	77	7	16	11	10	x	10	7	5	x	5	x	x
Q-shaped	*T*	10 nm				20 nm				30 nm				40 nm		
	Fano Modes	*F*_1_	*F*_2_	*F*_3_	*F*_4_	*F*_1_	*F*_2_	*F*_3_	*F*_4_	*F*_1_	*F*_2_	*F*_3_	*F*_4_	*F*_1_	*F*_2_	*F*_3_
	*Q*-factor	13	15	19	110	64	12	18	32	192	21	22	14	196	20	23
	FoM	5	8	6	33	32	6	10	13	68	14	16	8	105	14	17

**Table 4:** *Q*-factor and FoM values around Fano resonances.

*D*	Theta-Shaped: Double Split			Q-Shaped:Single Split			Q-Shaped: Double Split		
Sharp Fano Modes	5 nm	7 nm	9 nm	5 nm	7 nm	9 nm	5 nm	7 nm	9 nm
*Q*-factor	111	113	111	117	117	117	80	81	79
FoM	55	71	67	43	41	34	40	44	54

## References

[1] Giner-Casares J.J., Henriksen-Lacey M., Coronado-Puchau M., Liz-Marzán L.M. (2016). Inorganic nanoparticles for biomedicine: Where materials scientists meet medical research. Mater. Today.

[2] Xie T., Jing C., Long Y.-T. (2017). Single plasmonic nanoparticles as ultrasensitive sensors. Analyst.

[3] Gobin A.M., Lee M.H., Halas N.J., James W.D., Drezek R.A., West J.L. (2007). Near-infrared resonant nanoshells for combined optical imaging and photothermal cancer therapy. Nano Lett..

[4] Matsushita R., Kiguchi M. (2015). Surface enhanced Raman scattering of a single molecular junction. Phys. Chem. Chem. Phys..

[5] Kollatou T., Dimitriadis A., Assimonis S., Kantartzis N., Antonopoulos C. (2014). Multi-band, highly absorbing, microwave metamaterial structures. Appl. Phys. A.

[6] Monti A., Toscano A., Bilotti F. (2016). Exploiting the surface dispersion of nanoparticles to design optical-resistive sheets and Salisbury absorbers. Opt. Lett..

[7] Monti A., Toscano A., Bilotti F. (2017). Analysis of the scattering and absorption properties of ellipsoidal nanoparticle arrays for the design of full-color transparent screens. J. Appl. Phys..

[8] Mulla B., Sabah C. (2016). Multiband Metamaterial Absorber Design Based on Plasmonic Resonances for Solar Energy Harvesting. Plasmonics.

[9] Dayal G., Chin X.Y., Soci C., Singh R. (2017). High-Q Plasmonic Fano Resonance for Multiband Surface-Enhanced Infrared Absorption of Molecular Vibrational Sensing. Adv. Opt. Mater..

[10] Gupta M., Singh R. (2016). Toroidal versus Fano Resonances in High Q planar THz Metamaterials. Adv. Opt. Mater..

[11] Verellen N., Van Dorpe P., Vercruysse D., Vandenbosch G.A., Moshchalkov V.V. (2011). Dark and bright localized surface plasmons in nanocrosses. Opt. Express.

[12] Sun B., Zhao L., Wang C., Yi X., Liu Z., Wang G., Li J. (2014). Tunable Fano resonance in E-shape plasmonic nanocavities. J. Phys. Chem. C.

[13] Chen Z., Wang W., Cui L., Yu L., Duan G., Zhao Y., Xiao J. (2014). Spectral splitting based on electromagnetically induced transparency in plasmonic waveguide resonator system. Plasmonics.

[14] Khan A.D., Khan S.D., Khan R., Ahmad N., Ali A., Khalil A., Khan F.A. (2014). Generation of multiple Fano resonances in plasmonic split nanoring dimer. Plasmonics.

[15] Tamma V.A., Cui Y., Zhou J., Park W. (2013). Nanorod orientation dependence of tunable Fano resonance in plasmonic nanorod heptamers. Nanoscale.

[16] Deng Z.-L., Yogesh N., Chen X.-D., Chen W.-J., Dong J.-W., Ouyang Z., Wang G.P. (2015). Full controlling of Fano resonances in metal-slit superlattice. Sci. Rep..

[17] Deng Z.-L., Fu T., Ouyang Z., Wang G.P. (2016). Trimeric metasurfaces for independent control of bright and dark modes of Fano resonances. Appl. Phys. Lett..

[18] Deng Z.L., Li X., Fu T., Wang G.P. (2017). Fano resonance in a metasurface composed of graphene ribbon superlattice. IEEE Photonics J..

[19] Fang Z., Cai J., Yan Z., Nordlander P., Halas N.J., Zhu X. (2011). Removing a wedge from a metallic nanodisk reveals a Fano resonance. Nano Lett..

[20] Amin M., Khan A.D. (2015). Polarization Selective Electromagnetic-Induced Transparency in the Disordered Plasmonic Quasicrystal Structure. J. Phys. Chem. C.

[21] Luk’yanchuk B., Zheludev N.I., Maier S.A., Halas N.J., Nordlander P., Giessen H., Chong C.T. (2010). The Fano resonance in plasmonic nanostructures and metamaterials. Nat. Mater..

[22] Deng Z.-L., Dong J.-W. (2013). Lasing in plasmon-induced transparency nanocavity. Opt. Express.

[23] Singh R., Cao W., Al-Naib I., Cong L., Withayachumnankul W., Zhang W. (2014). Ultrasensitive terahertz sensing with high-Q Fano resonances in metasurfaces. Appl. Phys. Lett..

[24] Cong L., Manjappa M., Xu N., Al-Naib I., Zhang W., Singh R. (2015). Fano resonances in terahertz metasurfaces: A figure of merit optimization. Adv. Opt. Mater..

[25] Khan A.D., Miano G. (2013). Plasmonic Fano resonances in single-layer gold conical nanoshells. Plasmonics.

[26] Hu Y., Noelck S.J., Drezek R.A. (2010). Symmetry Breaking in Gold− Silica− Gold Multilayer Nanoshells. ACS Nano.

[27] Dayal G., Chin X.Y., Soci C., Singh R. (2016). High-Q Whispering-Gallery-Mode-Based Plasmonic Fano Resonances in Coupled Metallic Metasurfaces at Near Infrared Frequencies. Adv. Opt. Mater..

[28] Dayal G., Chin X.Y., Soci C., Singh R. (2016). Independent Tailoring of Super-Radiant and Sub-Radiant Modes in High-Q Plasmonic Fano Resonant Metasurfaces. Adv. Opt. Mater..

[29] Khan A.D., Khan S.D., Khan R.U., Ahmad N. (2014). Excitation of multiple Fano-like resonances induced by higher order plasmon modes in three-layered bimetallic nanoshell dimer. Plasmonics.

[30] Zhang W., Feng Y., Zhang Y., Chen W., Lin W. (2015). Sensitivity and tunability of heptamer clusters composed of asymmetric split nanorings. J. Phys. D Appl. Phys..

[31] Liu S.-D., Leong E.S.P., Li G.-C., Hou Y., Deng J., Teng J.H., Ong H.C., Lei D.Y. (2016). Polarization-independent multiple Fano resonances in plasmonic nonamers for multimode-matching enhanced multiband second-harmonic generation. ACS Nano.

[32] Binfeng Y., Hu G., Zhang R., Yiping C. (2016). Fano resonances in a plasmonic waveguide system composed of stub coupled with a square cavity resonator. J. Opt..

[33] Chen Z., Yu L., Wang L., Duan G., Zhao Y., Xiao J. (2015). Sharp asymmetric line shapes in a plasmonic waveguide system and its application in nanosensor. J. Lightw. Technol..

[34] Wu Y., Zheng H., Li J., Wang C., Li C., Dong J. (2015). Generation and manipulation of ultrahigh order plasmon resonances in visible and near-infrared region. Opt. Express.

[35] Verellen N., Van Dorpe P., Huang C., Lodewijks K., Vandenbosch G.A., Lagae L., Moshchalkov V.V. (2011). Plasmon line shaping using nanocrosses for high sensitivity localized surface plasmon resonance sensing. Nano Lett..

[36] Fu Y.H., Zhang J.B., Yu Y.F., Luk’yanchuk B. (2012). Generating and manipulating higher order Fano resonances in dual-disk ring plasmonic nanostructures. ACS Nano.

[37] Habteyes T.G., Dhuey S., Cabrini S., Schuck P.J., Leone S.R. (2011). Theta-shaped plasmonic nanostructures: bringing “dark” multipole plasmon resonances into action via conductive coupling. Nano Lett..

[38] Johnson P.B., Christy R.-W. (1972). Optical constants of the noble metals. Phys. Rev. B.

[39] Chen L., Xu N., Singh L., Cui T., Singh R., Zhu Y., Zhang W. (2017). Defect-Induced Fano Resonances in Corrugated Plasmonic Metamaterials. Adv. Opt. Mater..

[40] Baquedano E., González M., Paniagua-Domínguez R., Sánchez-Gil J., Postigo P. (2017). Low-cost and large-size nanoplasmonic sensor based on Fano resonances with fast response and high sensitivity. Opt. Express.

